# Purification and Characterization of Organic Solvent and Detergent Tolerant Lipase from Thermotolerant *Bacillus* sp. RN2

**DOI:** 10.3390/ijms11103783

**Published:** 2010-09-29

**Authors:** Pornpimon Kanjanavas, Sintawee Khuchareontaworn, Paisarn Khawsak, Arda Pakpitcharoen, Khajeenart Pothivejkul, Somchai Santiwatanakul, Kenji Matsui, Tadahiko Kajiwara, Kosum Chansiri

**Affiliations:** 1 Department of Biochemistry, Faculty of Medicine, Srinakharinwirot University, Bangkok 10110, Thailand; E-Mails: kanjanavas@hotmail.com (P.K.); wevansi@hotmail.com (S.K.); pkhla@hotmail.com (P.K.); oom_arda@hotmail.com (A.P.); 2 Department of Biology, Faculty of Sciences, Srinakharinwirot University, Bangkok 10110, Thailand; E-Mail: kajeenart@gmail.com; 3 Department of Pathology, Faculty of Medicine, Srinakharinwirot University, Bangkok 10110, Thailand; E-Mail: somchaii@swu.ac.th; 4 Department of Biological Chemistry, Faculty of Agriculture, Yamaguchi University, Yamaguchi, Japan; E-Mail: matsui@yamaguchi-u.ac.jp (K.M.)

**Keywords:** lipase, organic tolerant, detergent tolerant, *Bacillus*, thermotolerant

## Abstract

The aim of this study was to characterize the organic solvent and detergent tolerant properties of recombinant lipase isolated from thermotolerant *Bacillus* sp. RN2 (Lip-SBRN2). The isolation of the lipase-coding gene was achieved by the use of inverse and direct PCR. The complete DNA sequencing of the gene revealed that the *lip-SBRN2* gene contains 576 nucleotides which corresponded to 192 deduced amino acids. The purified enzyme was homogeneous with the estimated molecular mass of 19 kDa as determined by SDS-PAGE and gel filtration. The Lip-SBRN2 was stable in a pH range of 9–11 and temperature range of 45–60 °C. The enzyme was a non metallo-monomeric protein and was active against *p*NP-caprylate (C8) and *p*NP-laurate (C12) and coconut oil. The Lip-SBRN2 exhibited a high level of activity in the presence of 108% benzene, 102.4% diethylether and 112% SDS. It is anticipated that the organic solvent and detergent tolerant enzyme secreted by *Bacillus* sp. RN2 will be applicable as catalysts for reaction in the presence of organic solvents and detergents.

## 1. Introduction

Lipases (glycerol ester hydrolases EC 3.1.1.3) are an industrially important subgroup of the α/β hydrolase superfamily [1]. They are used in fat hydrolysis or as catalysts in synthetic organic chemistry where their regiospecificity and enantioselectivity are desired characteristics [2]. Although lipases belong to many different protein families without sequence similarity, they have the same architecture, the α/β hydrolase fold [3] and a conserved active site signature, the GxSxG motif [4,5]. They all share the same catalytic machinery consisting of a catalytic triad (serine-histidine-aspartic/glutamic acid) and oxyanion hole, formed by the backbone amides of two conserved residues. Ester hydrolysis is mediated by a nucleophilic attack of the active serine on the carbonyl group of the substrate, in a charge-relay system with the two other amino acid residues of the catalytic triad [2]. In nonaqueous systems, lipases catalyze the reverse reaction, namely ester synthesis and trans-esterification. Therefore, there is an increasing interest for lipases and lipase-producing strains for biotechnological applications, as lipasecatalyzed reactions show high selectivity and occur under mild conditions, with no requirement for added cofactors [6].

The interest in bacterial lipases has increased due to the fact that they are more stable than those from other organisms, especially when exposed to high temperature or other severe conditions. Thermotolerant *Bacillus* species are among those micro-organisms capable to promote lipid conversion by means of their lipolytic systems and high stability under such conditions. Several lipases from thermotolerant *B. licheniformis* [7], *B. thermocatenulatus* [8], *B. thermoleovorans* [9], or *B. stearothermophilus* [10] have already been described, cloned or purified. The increasing interest for thermotolerant lipase-producing *Bacillus* strains led us to perform the analysis of *Bacillus* sp. RN2 lipase. The organic solvent and detergent tolerant properties of the enzyme could be evaluated and used in certain manufacturing processes such as antibiotic production [11] biosurfactants and biofilm production [12–14] biodegradation of recalcitrant substances [15], or in the conversion of low-cost fats into value-added products [6].

## 2. Results and Discussion

### 2.1. Isolation and Characterization of *Bacillus* sp. RN 2 Lipase

The isolate RN2 was chosen for a subsequent experiment due to its high activity in lipase production. The isolate RN2 was gram positive bacteria, an aerobic and rod-shape, which could grow within a temperature range of 40–75 °C with an optimum at 50 °C. The molecular classification of the isolate based on the hypervariant region (HV) of the 16S rDNA sequence (accession no. HQ112249) exhibited a similarity of 98% to *B. licheniformis* accession no. DQ923804.

**Figure 1:**
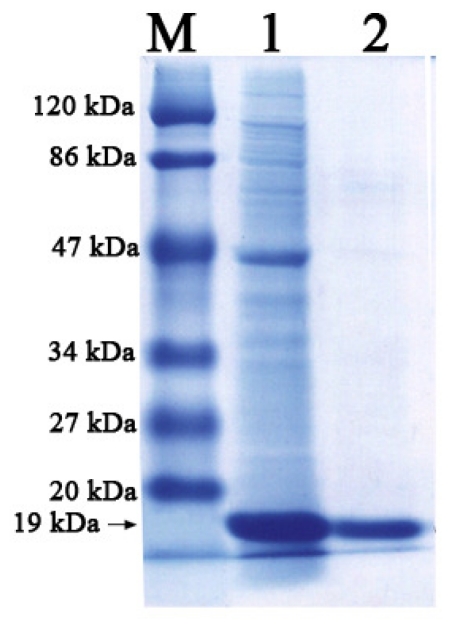
SDS-PAGE profile of Lip-SBRN2. Lane M represents protein marker, lane 1 represents crude lipase and lane 2 represents purified Lip-SBRN2.

The *lip-SBRN2* gene (accession no. EF584562) was composed of 576 nucleotides that coded for 192 amino acids with a molecular mass of approximately 19 kDa (Figure 1). The catalytic apparatus of the *lip-SBRN2* gene, involving the triad serine, glutamate or aspartate and histidine, was placed in the newly isolated lipase at positions Ser77, Asp129 and His152. The common motif AHSMG and oxyanion hole PVVMVHG regions of all known mesophilic *Bacillus* lipases were found in the protein, with the first glycine typical residue being replaced by an alanine. The results obtained from amino acid sequence homology analysis indicated that the *lip-SBRN2* gene is a member of a reduced cluster of highly conserved bacterial serine-esterases grouped (AHSMG) and oxyanion hole (PVVMVHG) in subfamily 1.4, exclusive from the mesophilic or moderately thermophilic members of the genus *Bacillus* [16]. The deduced amino acid sequences also indicated that Lip-SBRN2 is synthesized as a pre-protein with an amino-terminal signal peptide sequence. This signal peptide is needed for proper targeting of the protein, and is removed before the mature protein is released into the external fluid.

### 2.2. Expression and Purification of Lip-SBRN2

Purification of Lip-SBRN2 using Ni-NTA column gave 51% yield with a specific activity of 118 units (Table 1). SDS-PAGE revealed that the purified Lip-SBRN2 enzyme possessed the relative molecular weight of approximately 19 kDa, which is similar to lipases of *B. subtilis* and *B. pumilus* [7,16]. It is notable that these group of lipases lack cysteine amino acid residues, indicating the absence of stabilizing disulfide bridges. This was confirmed by the retention of Lip-SBRN2 activity in the presence of reducing agents, DTT and β-mercaptoethanol.

### 2.3. Optimum pH and Temperature for Lip-SBRN2 Activity

**Figure 2:**
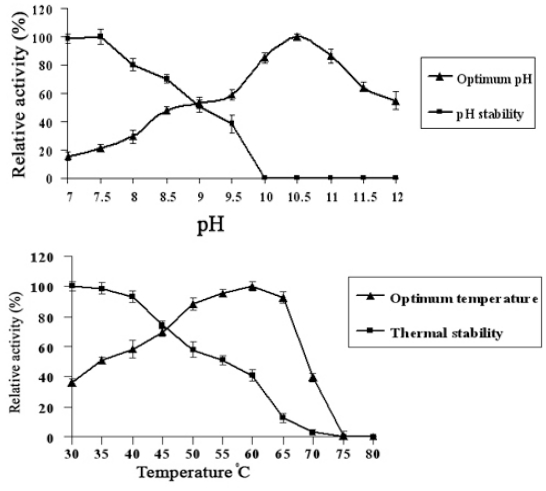
(**A**) The effect of pH and pH stability on Lip-SBRN2 activity was determined by spectrophotometric assay of *p*-nitrophenyl laurate (*p*NPL). The substrate was prepared in 50 mM buffer volumes of various pH values. The following buffers were used: potassium phosphate buffer (pH 7–8.5), Tris-HCl (pH 9–10.5) and glycine/NaOH (pH 11–12). The pH stability was determined by incubating enzyme at various pH at room temperature for 4 h and assay at optimum temperature. (**B**) The thermal stability and optimum temperature for Lip-SBRN2 activity was determined by spectrophotometric assay of *p*NPL. The assay mixture was equilibrated at the temperature range of 30–80 °C before adding the enzyme. The effect of temperature stability was determined by incubating the enzyme for 30 min at various temperatures. All reactions were performed at least in duplicate.

In stability assays, the Lip-SBRN2 displayed maximal catalytic activity towards *p*-nitrophenyl laurate (*p*NPL) at pH 10.5 and retained 100% activity after incubation at pH 7–7.5 for 4 h (Figure 2A). The enzyme exhibited optimum activity at temperatures in the range of 55–65 °C with maximal activity at 60 °C after incubation for 30 min. The stability test revealed that the residual enzyme activity was 90–100% after incubation at 30–40 °C for 30 min (Figure 2B). The data indicate that the Lip-SBRN2 is a mesophilic enzyme acting at a broad range of pH and temperature, which corresponded to the features of true lipase in subfamily 1.4.

### 2.4. Effect of Metal Ions and Chemical Reagent on Lip-SBRN2 Activity

**Figure 3:**
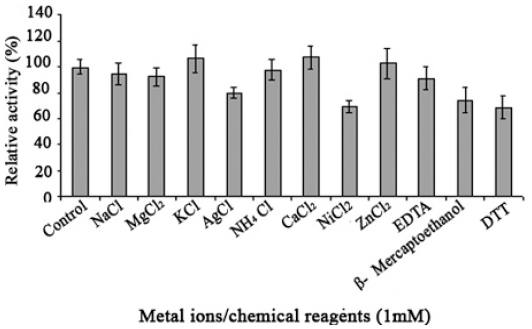
Effect of metal ions and inhibitors on Lip-SBRN2 activity. The enzyme (1.3 mg/mL) was pre-incubated with 1 mM of each reagent listed at 45 °C for 30 min. The residual enzyme activity in comparison to control was determined using lipase assay. All reactions were performed at least in duplicate.

The effect of different metal ions and inhibitors on Lip-SBRN2 activity was determined using *p*NPL as a substrate (Figure 3). The effect of metal ions revealed that no significant inhibition of Lip-SBRN2 activity was obtained. In addition, the lipase activity was not significantly inhibited by EDTA, DTT, SDS and β-mercaptoethanol. In the presence of EDTA, the enzyme could retain almost 90% activity. These data implied that the biochemical properties of the recombinant lipase are the same as those of true lipases subfamily 1.4 as they are non-metallo monomeric proteins with the absence of disulfide bonds in the structure, suggesting that this protein assumes a flexible tertiary structure which may facilitate conformational change.

### 2.5. Substrate Specificity

The highest hydrolysis rates of Lip-SBRN2 were obtained with *p*NP laurate (100%) and coconut oil (727.3%), as shown in Table 2. The Lip-SBRN2 displayed a substrate profile similar to those found for most esterases, showing higher affinity for medium chain-length substrates [2]. The lipase showed the highest activity towards *p*-nitrophenyl laurate (C12). This indicated the enzyme’s preference for medium-size acyl chain length substrates. However, the enzyme could also hydrolyze long chain substrates, e.g., *p*NP myristate (C14), *p*NP palmitate (C16) and *p*NP stearate (C18), with the relative activity of about 45%. The presence of good lipase activity towards medium and long-chain fatty acid esters indicated that it is a true lipase in contrast to esterase that hydrolyzes exclusively short chain fatty acyl esters.

### 2.6. Effect of Organic Solvents and Detergent on Lip-SBRN2 Activity

Exposure of the Lip-SBRN2 to various organic solvents for 30 min showed that this enzyme retained activity in all organic solvent tested (Table 3). The highest relative activity was achieved at 108.0%, 102.4%, and 100.8% in benzene, diethylether, and acetone, respectively. However, the activities were slightly decreased when the enzymes incubation were extended to 2 h in benzene and acetone. In contrast, the enzyme activity was increased in dimethylether, n-hexane, cyclohexane, N,N-diethylformaminde, chloroform and 2-propanol after incubated for 2 h in the same conditions. The stability of Lip-SBRN2 in aqueous-organic mixtures suggested the ability of this enzyme to retain activity in organic solvents and held the potential for its use in organic synthesis and related applications. This data was in contrast to the lipase of *Psuedomonas cepacia* which was inactive at all concentration of benzene and hexane after 30 min incubation [20]. The efficiency of Lip-SBRN2 in the presence of detergents was dramatically increased especially when it was incubated with 1% SDS for 2 h using olive oil as a substrate. SDS could improve the oil substrate solubility in water, which assisted the activity of the enzyme on lipid hydrolysis.

## 3. Experimental Section

### 3.1. Bacterial Strains Collection

Thermotolerant *Bacillus* spp. were isolated from hot spring water at Ranong province of Thailand. Soil and water samples were screened for lipase activity by observing the clear zone on tributyrin agar. Chromosomal DNA of *Bacillus* sp. RN2 was isolated and purified using a DNA extraction kit (QIAGEN). For the amplification and sequencing of the 16S rDNA-HV region, two primers were constructed based on the result of multiple alignments of 16S rRNA sequences from 69 bacillus type strains using CLUSTAL W version 1.74 as described by Goto and colleagues [21].

### 3.2. PCR Amplification of Thermotolerant *Bacillus* sp. RN2 Lipase Gene (Lip-SBRN2 Gene)

The *lip-SBRN2* gene was obtained by PCR using the forward primer 5′ ATT CTA TTG ATT TGC ATG CTG TCT G 3′ and the reverse primer 5′ CCT TGA AGA AGT TAA GCT CTT CAA G 3′. The PCR condition contained one cycle of 95 °C for 2 min followed by an additional 30 cycles of denaturation at 94 °C for 45 s, annealing at 57 °C for 1 min and extension at 72 °C for 1 min. The post extension was carried on for another 10 min at 72 °C. Amplified PCR fragment was purified through MinElute Gel Extraction Kit (QIAGEN), ligated to pGEM T easy vector (BIO-RAD), transformed into *Escherichia coli* XL1-Blue by electroporation (BIO-RAD), and sequenced using BigDye™ Terminator V 3.0 Cycle Sequencing Ready Reaction. The nucleotide and amino acid alignments used ClustalW (http://www.ebi.ac.uk) and SignalP V2.0 program, respectively.

### 3.3. Over-Expression of the Lip-SBRN2 Gene

The *lip-SBRN2* gene was ligated into the pET 100/D TOPO^®^vector using Champion™ pET Directional TOPO^®^ expression kit (Invitrogen, Gibco BRL). The recombinant DNA was transformed into *E. coli* BL21 star ™ (DE3) one shot^®^ cell (Gibco BRL) using electroporation. The positive transformants were selected and tested for lipase activity. *E. coli* BL21 cells transformed with *lip-SBRN2* were grown in 1 L of LB medium at 37 °C containing 100 μg of ampicillin until an OD of 0.5 at 600 nm was attained. Lipase activity was induced by addition of isopropyl-β-d-thiogalactopyranoside (IPTG) to a final concentration of 0.1 mM and incubated for 5 h at 37 °C.

### 3.4. Purification of Lip-SBRN2

The cultured solution was centrifuged at 6,000 × g for 10 min. The bacterial pellets were resuspended with binding buffer (50 mM NaH_2_PO_4_ [pH 8], 300 mM NaCl, 10 mM imidazole) and disrupted by sonication. Cell debris was removed by centrifugation at 6,000 × g for 10 min and the supernatant was purified by using Ni-NTA column chromatography (QIAGEN). The fractions containing lipase activity were pooled and protein concentration determined using Bradford assay [22]. The molecular weight of protein was estimated using the standard protocol of 12.5% SDS-PAGE [23].

### 3.5. Lipase Assay

Two procedures were used for the determination of Lip-SBRN2 activity. The first method relied on spectrophotometric assay using *p*-nitrophenyl laurate (*p*NPL) as a substrate [21,22]. One unit of lipase activity is defined as the amounts of enzyme releasing 1 μmol *p*NP per minute under the assay conditions. The other was based on determination of liberated free fatty acid (FFA) using the method of Kwon and Rhee [23]. One unit of lipase activity is the amount of enzyme that liberated 1 μM of free fatty acid in 1 min at 37 °C. The effect of pH on Lip-SBRN2 activity was determined. The substrate was prepared in 50 mM phosphate buffer (pH 7–8.5), Tris-HCl (pH 9–10.5) and glycine/NaOH (pH 11–12). The optimum temperature for Lip-SBRN2 activity was determined with *p*NPL in the temperature range of 30–80 °C.

## 4. Conclusion

The stability of Lip-SBRN2 in some organic solvents and detergents as well as the retained activity in the presence of some metals ions and inhibitors suggested that the enzyme could be exploited in certain manufacturing processes such as antibiotic production, biosurfactant and biofilm production, biodegradation of recalcitrant substances, or in the conversion of low-cost fats into added value products.

## Figures and Tables

**Table 1:** Purification of Lip-SBRN2.

Steps	Volume (mL)	Total activity (Units)	Total activity/mL (Units/mL)	Total protein (mg)	Specific activity (Units/mg)	Purification factor (fold)	Recovery yield (%)
Crude enzyme	5	281	56.2	174	1.6	1	100
Ni-NTA column	2	144	72	1.3	118	74	51

**Table 2:** The substrate specificity of Lip-SBRN2 using various *p*-nitrophenylesters was assayed spectrophotometrically as previously described [17,18]. The substrates were dissolved in isopropanol at a concentration of 10 mM. The results are expressed as percentage of the substrate that generates the maximal activity (*p*NP-laurate (C12) as a control). Determination of liberated free fatty acid (FFA) from natural oils substrates was assayed by using the method of Kwon and Rhee [19] (olive oil (18:1) as a control). All reactions were performed at least in duplicate.

Substrates	Relative activity (%)
**p-nitrophenylester**	
pNP-butyrate (C4)	40 ± 6
pNP-caprylate (C8)	92 ± 8
pNP-caprate (C10)	68 ± 9
pNP-laurate (C12)	100 ± 15
pNP-myristate (C14)	59 ± 7
pNP-palmitate (C16)	54 ± 10
pNP-stearate (C18)	35 ± 4
**Natural oils**	
Coconut oil (12:0)	727.3 ± 16
Palm oil (16:0)	32.0 ± 13
Olive oil (18:1)	100.0 ± 9
Grape seed oil (18:2)	32.4 ± 7
Soybean oil (18:3)	42.2 ± 7

**Table 3:** Stability of Lip-SBRN2 in various organic solvents and detergents were determined using olive oil as a substrate. The residual activity of Lip-SBRN2 was determined by incubation of 3 mL of enzyme in L ml of organic solvents. The reaction was carried out in a 15 mL screw cap tube, mixed and incubated at 37 °C with agitation at 150 rpm for 30 min and 2 h. After that, 1 mL of enzyme-organic solvent mixture was mixed with 2.5 mL of olive oil emulsion in the presence of 0.02 mL of 20 mM CaCl_2_·2H_2_O. The assay was continued according to the method of Kwon and Rhee [19]. All reactions were performed at least in duplicate.

Organic solvents/detergents (25%)	Relative activity of *Bacillus* sp. RN2 lipase	
	30 min incubation Relative activity (%)	2 h incubation Relative activity (%)
None	100.0 ± 7	100.0 ± 4
**Organic solvents**		
Benzene	108.0 ± 11	104.4 ± 3.4
Diethylether	102.4 ± 5.2	103.6 ± 0.5
Acetone	100.8 ± 0.8	82.2 ± 11.3
n-hexane	94.0 ± 4	94.5 ± 3.5
Dimethylsulfoxide	85.2 ±.8.4	96.4 ± 11.3
Cyclohexane	84.4 ± 11.2	85.1 ± 5.6
*N*,*N*-dimethylformamide	80.4 ± 5.5	86.9 ± 3.4
Chloroform	80.0 ± 11	84.4 ± 10.5
Acetonitrile	69.6 ± 9.5	82.5 ± 11
Toluene	68.4 ± 12.3	80.4 ± 13.4
2-propanol	60.8 ± 8.7	66.5 ± 6.6
**Detergents**		
25%Tween-20	60.8 ± 11.5	96.4 ± 7.3
25%Triton X-100	41.6 ± 10.6	86.5 ± 8.7
1% SDS	112.5 ± 11.3	485.7 ± 11.5
